# Enzymatic cleavage of model lignin dimers depends on pH, enzyme, and bond type

**DOI:** 10.1038/s41598-025-88571-7

**Published:** 2025-03-25

**Authors:** Jenny R. Onley, Kshitiz Gupta, Markus de Raad, Benjamin P. Bowen, Stephen Tan, Sam Yoder, Kenneth L. Sale, Anup K. Singh, Blake A. Simmons, Paul D. Adams, Trent R. Northen, Kai Deng

**Affiliations:** 1 Technology Division, Joint BioEnergy Institute, Emeryville, CA USA; 2 Biomaterials and Biomanufacturing Department, Sandia National Laboratories, Livermore, CA USA; 3 Engineering Directorate, Lawrence Livermore National Laboratory, Livermore, CA USA; 4 Environmental Genomics and Systems Biology Division, Lawrence Berkeley National Laboratory, Berkeley, CA USA; 5 Biological Systems and Engineering Division, Lawrence Berkeley National Laboratory, Berkeley, CA USA; 6 Biosecurity and Bioassurance Department, Sandia National Laboratories, Livermore, CA USA; 7 Deconstruction Division, Joint BioEnergy Institute, Emeryville, CA USA; 8 Molecular Biophysics and Integrated Bioimaging, Lawrence Berkeley National Laboratory, Berkeley, USA; 9 Department of Bioengineering, University of California, Berkeley, USA

**Keywords:** Lignin, Laccase, Peroxidase, Mass spectrometry, Biochemistry, Analytical biochemistry, High-throughput screening, Mass spectrometry

## Abstract

Lignin is composed of phenylpropanoid monomers linked by ether and carbon-carbon bonds to form a complex heterogeneous structure. Bond-specific studies of lignin-modifying enzymes (LMEs; e.g., laccases and peroxidases) are limited by the polymerization of model lignin substrates and repolymerization of cleavage products. Here we present a high throughput platform to screen LME activities on four tagged model lignin compounds that represent the β-O-4’, β-β’, 5–5’, and 4-O-5’ linkages in lignin. We utilized nanostructure-initiator mass spectrometry (NIMS) and model lignin compounds with tags containing perfluorinated and cationic moieties, which effectively limit polymerization and condensation of the substrates and their degrading products. Sub-microliter sample droplets were printed on the NIMS chip with a novel robotics method. This rapid platform enabled characterization of LMEs across a range of pH 3–10 and relative quantification of modified (typically oxidized), cleaved, and polymerized products. All tested enzymes oxidized the four substrates and cleaved the β-O-4’ and β-β’ substrates to monomeric products. We discovered that the active pH range depended on both the substrate and the enzyme type. This has important applications for biomass conversion to biofuels and bioproducts, where the relative percentages of different bond types in lignin varies depending on feedstock and chemical pretreatment methods.

**Supplementary Information:**

The online version contains supplementary material available at 10.1038/s41598-025-88571-7.

## Background

Although lignocellulose is the most abundant source of renewable carbon on earth^1^, only 2% of lignin from the pulp and paper industry is utilized commercially^2^. Harsh chemical treatments show great promise for lignin depolymerization, but the poor recyclability of solvents, low product yield, and the limited bioavailability of depolymerized lignin streams for conversion to products by engineered host organisms continue to limit the commercial application of these processes^3,4^. Enzymatic depolymerization of lignin with lignin-modifying enzymes (LMEs) is considered a green alternative to harsh chemical processes^5,6^. LMEs include laccases, versatile peroxidases, lignin peroxidases, and manganese peroxidases, which apply radical chemistry to break the bonds in lignin. Laccases and peroxidases are used by both fungi and bacteria to depolymerize lignin in nature^7,8^. Fungal LMEs such as laccases typically have pH optima ranging from acidic to neutral^8^. Biomass pretreatment methods^9,10^and catalytic lignin depolymerization methods^4,11^include acidic and basic treatments, so efforts have been made to enhance the activity of LMEs at both low^12^and high^13,14^ pH extremes. Understanding the pH optima of LMEs is crucial for their application in lignocellulose deconstruction and lignin valorization.

Decades of studying LMEs^15,16^have revealed the radical mechanisms LMEs use to polymerize and cleave aromatic monomers and dimers. However, we still have a poor understanding of how LMEs cleave the different interunit bonds in lignin. One major factor is the lack of analytical tools for the characterization of LMEs. The strengths and weaknesses of approaches for testing LMEs were recently reviewed^17^. The most common techniques for LME characterization are spectrophotometric assays with synthetic compounds such as 2,2′-azino-bis(3-ethylbenzthiazoline-6-sulfonic acid) (ABTS), or with phenolic monomers such as 2,6-dimethoxyphenol, guaiacol, and syringaldehyde^8,17^. The optimal pH for LMEs differs depending on which substrate is tested, and spectrophotometric assays provide limited information regarding activity with aromatic polymers.

**Fig. 1:**
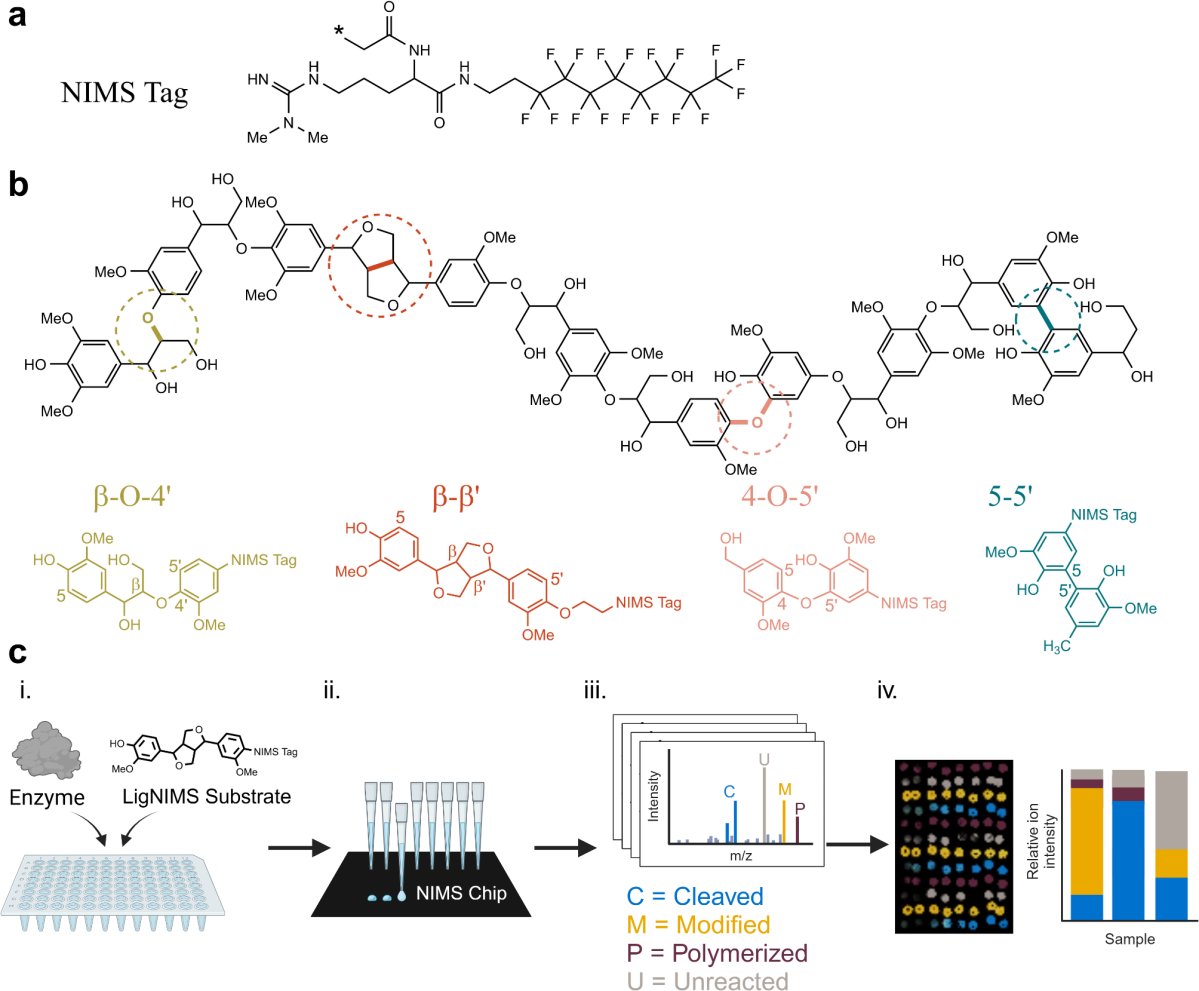
**a** Chemical structure of the NIMS tag. Asterisk indicates the attachment point for the model lignin compound. **b** Hypothetical structure of lignin (central structure in black) with common bond types highlighted by colored circles and colored bonds. The structure of the NIMS-tagged model lignin compounds (“LigNIMS substrates”) are shown by the colored structures. The hypothetical lignin structure is intended for illustrative purposes and is a simplified representation of true lignin structure (for example, most 5–5’ bonds are located in dibenzodioxocin structures, which are not pictured here, and relative proportions of bond types do not reflect natural ratios). **c** Workflow for rapid testing of enzymes with the LigNIMS substrates. **i** Enzymatic reactions were prepared in 96-well PCR plates via robotic liquid handlers and/or multichannel pipettes. **ii** Samples were acidified, then transferred to the NIMS chip with robotic liquid handlers by touching the NIMS chip surface with the pipette tips. **iii** Dried droplet arrays were analyzed by mass spectrometry imaging. **iv** Sample locations were determined via the OpenMSI Arrayed Analysis Toolkit (OMAAT) and ions were binned according to their *m/z* values to either “cleaved,” “unreacted,” “modified,” or “polymerized.” The relative ion intensities of these four categories were plotted. Figure created in part with ChemDraw (a-b) and biorender.com (c).

LME characterization with native or pretreated lignin would be more ideal for biorefinery applications, but structural analysis of lignin is time consuming and cannot provide data that is detailed enough for quantification of bond breaking events or percent yield of breakdown products. Lignin is a complex heteropolymer composed of phenylpropanoid monomers linked together by ether and carbon-carbon linkages, including β-O-4’, β-β’, 4-O-5’, and 5–5’ linkages (Fig. 1b). Lignin is insoluble in water, further complicating analytical characterization. Moreover, the degradation products tend to condense or to couple with other lignin fragments to make more complex structures. The current most common technique to characterize modifications of lignin (including interunit bond cleavage) is two-dimensional heteronuclear single quantum coherence nuclear magnetic resonance (2D-HSQC-NMR)^18–20^. While 2D-HSQC-NMR can identify the types of bonds being modified, it has limitations, including low sensitivity^19^, the need for large sample quantities (> 100 mg), lengthy data acquisition times (12 h or more), and a lack of detailed mechanistic information. Analytical methods that provide more granular data are required to truly understand the interactions of LMEs with the different bond types in lignin.

Another approach for researching enzymatic lignin degradation is to test lignin dimers that are interconnected by the same types of bonds found in lignin; for example, guaiacyl-glycerol-β-guaiacyl ether (G-O-4’) to represent β-O-4’ bonds and pinoresinol to represent β-β’ bonds. LMEs polymerize G-O-4’ and pinoresinol^21 ^but generally cleave the syringyl forms of these compounds (syringyl-glycerol-β-guaiacyl ether [S-O-4’] and syringaresinol, respectively)^22,23^. Studies with versatile peroxidase suggest that cleavage of the guaiacyl form is possible in some conditions, and that the occupation of the 5 carbon (i.e., guaiacyl versus syringyl form) and pH determine relative activity and polymerization versus cleavage^24^. While these studies demonstrate the reactions between LMEs and lignin-derived dimers, they do not necessarily represent the reactions LMEs would catalyze on interunit bonds within the broader structure of lignin. Additionally, lignin dimers can be expensive and difficult to source, limiting extensive characterization of enzymes with dimers. An alternative approach is to synthesize tagged lignin dimers for nanostructure-initiator mass spectrometry (NIMS)^25^. The perfluorinated NIMS tag (Fig. 1a) facilitates the formation of micelle structures, effectively limiting the polymerization of the substrates. In comparison to mass spectrometry coupled with liquid chromatography, where analysis of lignin dimers requires extraction and lengthy (10 min per sample) methods^26^, NIMS requires only nanoliter sample volumes and can be analyzed quickly (seconds to minutes per sample), allowing for rapid screening of different enzymes and conditions^27,28^ and evaluation of enzyme kinetics^29^. Past experiments with NIMS-tagged G-O-4’ (the guaiacyl form of the β-O-4’ linkage) demonstrated that laccases, a lignin peroxidase, and a manganese peroxidase cleave the Cɑ-alkyl phenyl (C-C) bond and the β-ether (C-O) bonds^12,25,30,31^. Intermediate oxidation and final cleavage products followed a reaction pathway similar to the cleavage of S-O-4’^22^ demonstrating that NIMS can reveal potential reaction mechanisms for guaiacyl-form substrates when polymerization is limited by the micelle structure formation of the NIMS substrates.

Although the β-O-4’ bond makes up 45–94% of the interunit bonds in lignin^32,33^, other bond types are enriched in lignin after chemical pretreatment^34^. Therefore, it is important to understand how different interunit bond types are cleaved. In this study, our goal was to develop and use surrogate substrates for the major lignin bond types in a way that enables high throughput studies similar to plate-based assays, but with more chemical specificity and limited polymerization. Additionally, we evaluated the effects of pH on LME activity on different bond types. We deployed a suite of four NIMS-tagged model lignin substrates: the previously-published β-O-4’ substrate^25^ and three new substrates representing β-β’, 4-O-5’, and 5–5’ linkages (Fig. 1b). We evaluated five laccases (three fungal and two bacterial) and five horseradish peroxidases at a range of pH 3 to 10 and demonstrated that the pH optima depend on enzyme and on the bond type. The β-O-4’ and β-β’ substrates were oxidized then cleaved by all tested enzymes. We proposed a pathway for β-β’ cleavage and demonstrated that pH influences the relative abundance of cleavage products. Most products from the 5–5’ and 4-O-5’ reactions were oxidation products, with limited cleavage. In many cases, the optimal pH range for an enzyme varied depending on the substrate. The insights into cleavage mechanisms for different bond types at different pH have implications for the carbon cycle and for lignin valorization.

## Methods

### Chemicals

**Fig. 2:**
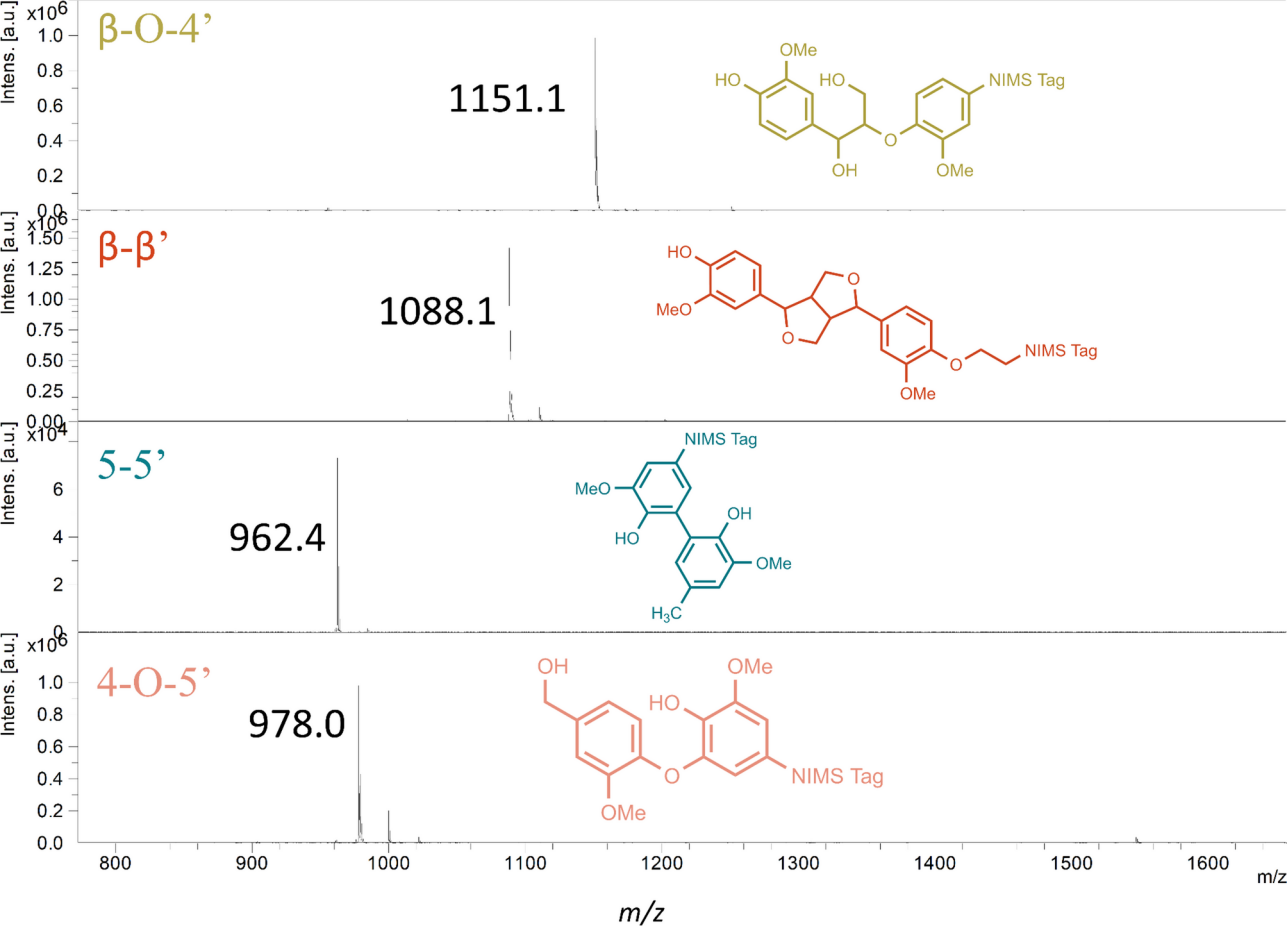
Mass spectra of the synthesized LigNIMS compounds. The y-axis shows intensity (arbitrary units). The β-O-4’ substrate was synthesized with a different, previously designed tag structure^25^. Measured *m/z* values for all four LigNIMS substrates are shown.

Sodium acetate, sodium phosphate dibasic, sodium carbonate, sodium bicarbonate, H_2_O_2_, and trifluoroacetic acid were sourced from Sigma-Aldrich (St. Louis, MO, USA). Glacial acetic acid was purchased from VWR. Citric acid (10% solution) was purchased from RICCA. 1-hydroxybenzotriazole monohydrate was purchased from TCI (Tokyo Chemical Industry Co., Ltd.). *Aspergillus* sp. laccase (cat. # SAE0050-50mL), *Trametes versicolor* laccase (cat. # 38429-1G), *Agaricus bisporus* laccase (cat. # 40452-100MG) and five horseradish peroxidases (P8125, P8250, P6782, P8375, and P8415) were purchased from Sigma-Aldrich. “*Aspergillus* sp. laccase” contains a *Myceliophthora thermophila* laccase that is expressed in *Aspergillus oryzae*^35^. Metzyme bacterial laccases L111 and L371^13^ were provided by MetGen Oy (Kaarina, Finland, www.metgen.com). Metzyme L371 is engineered to be alkaliphilic. NIMS-tagged substrates (Fig. 2) were synthesized and purified in house. Details about the synthetic scheme and characterization of new compounds can be found in Supplementary Figure S1 and the Supplementary Methods.

### Enzyme activity screen with LigNIMS

Dry commercial enzymes were prepared at a concentration of 0.5 mg/mL in nanopure water. The *Aspergillus* sp. laccase was diluted 1:20 in nanopure water. Metzyme laccases were diluted 1:100 in nanopure water, centrifuged, and the supernatant was tested. Enzyme reactions were prepared in 96-well PCR plates (Avantor cat. # 82006-636) with manual pipetting and liquid handling robots; Mantis^®^ Liquid Dispenser (Formulatrix^®^, Dubai, United Arab Emirates), the Biomek NX Liquid handler (Beckman Coulter, Brea, California, USA), and the Microlab Vantage (Hamilton, Reno, Nevada, USA) (Fig. 1c i). Laccases couple the oxidation of aromatic compounds to the reduction of O_2_ to H_2_O via the generation of free radicals^36^. Horseradish peroxidases require H_2_O_2_ for radical generation and aromatic oxidation, so H_2_O_2_ was included in all horseradish peroxidase incubations. Laccase reactions consisted of 2 µL enzyme, 2 µL of 0.5 mM NIMS substrate, and 6 µL buffer. Horseradish peroxidase incubations contained 2 µL enzyme, 2 µL of 0.5 mM NIMS substrate, 2 µl of 0.15% H_2_O_2_ and 4 µl buffer. A final concentration of 1 mg/mL *T. versicolor* laccase was used for the time series in Fig. 4a. Reactions contained undiluted McIlvaine’s (citrate-phosphate) buffer (pH 3–6), 0.1 M phosphate buffer (pH 7 and 8), or 0.1 M bicarbonate buffer (pH 9 and 10). Experiments with mediator included a 1:5 dilution of a 10 mM 1-hydroxybenzotriazole stock in 3% DMSO for a final concentration of 2 mM 1-hydroxybenzotriazole and 0.6% DMSO. Assays were set up in 96-well PCR plates, sealed with foil seals (Bio-Rad cat # MSF1001) and incubated at 30 °C (60 °C for Metzyme laccases, except for mediator study) for three hours. Samples were mixed 1:1 with 0.2% trifluoroacetic acid before transferring to the NIMS chip. Unless otherwise noted, experiments were set up in three or four independent repeat experiments.

### High-throughput NIMS printing

The analytes were deposited on the NIMS silicon chip using liquid handling robots such as Hamilton Microlab Vantage and Beckman-Coulter Biomek NX (Fig. 1c ii). A novel contact-based deposition method was developed for spotting the droplets. 20-µL pipette tips were dipped with a liquid handling robot in a 96-well source plate to wick a minimal amount of the analyte samples. The liquid was transferred by gently touching the pipette tip on the NIMS chip surface. The chip was fastened on a spring-loaded adapter which compresses and allows the pipette tip to make a soft contact with it. An 8-channel pipetting arm simultaneously aspirated 8 samples and then deposited droplets on the chip to improve the throughput. Analyte droplets were deposited in an 8 × 12 array in which adjacent droplets were 1.4 mm apart from each other. Overlap between sample spots was minimal (Supplementary Figure S2). The liquid viscosity, its surface tension with the pipette tip and the NIMS chip, and the pipette tip bore size primarily governed the quantity of the analyte dispensed. We used 20 µL tips with a bore size of approximately 0.6 mm which transfer sub-microliter droplets. We estimated the order of the volume dispensed by assuming the droplets are hemispherical in shape and measuring their diameter with microscope images. Although we did not have as precise control over the volume dispensed as some commercial droplet dispensers (Dispendix iDot or Labcyte Echo) our technique had a much higher resolution in arraying the sample droplets. We found both iDot and Echo to be unreliable in creating droplet arrays denser than a 1536-well format (approximately 222 samples/cm^2^). Whereas our technique created a droplet array which was 2.6-times as dense as a 1536-well plate (approximately 568 samples/cm^2^). Since we compared the signal intensities of different analytes within each sample spot to determine relative abundance, we did not need as precise a control on the volume dispensed.

### Mass spectrometry imaging (MSI) and data analysis

MSI was performed using the Bruker ultrafleXtreme MALDI-TOF mass spectrometer over a mass range of 200–3500 Da and a sample rate of 1.25 GS/s (Fig. 1c iii). Each position accumulated 400 laser shots. The instrument was controlled using the Bruker flexControl and Bruker flexImaging software. The imaging method included a 125 µm step size between shots. Spectra were recorded in positive reflector mode. The instrument was calibrated using Anaspec Peptide Calibration mixture 1 (catalog #AS-60882; Anaspec, Fremont, CA). Spectra were exported without binning. A modified version of the OpenMSI Arrayed Analysis Toolkit (OMAAT)^37^ identified sample spots (Fig. 1c iv) and exported all the spectral data for each sample spot. The terminology defined in de Raad et al.^37^ is employed in this paper: a sample spot refers to the physical sample on the NIMS chip surface, and a pixel refers to a single position in the MSI image raster. Peaks were determined via peakdet (http://billauer.co.il/peakdet.html) with a peak delta (i.e., the difference in intensity between the peak maximum value and the preceding minimum value) threshold of 10 and keeping 5 top peaks per pixel analyzed. Due to the irregularly shaped sample spots (a common issue with the dried droplet method), pixels that lacked signals with a relative signal intensity of approximately ten times above background noise were excluded. Any peak with a value less than ten times above background was excluded (i.e., intensity value set to zero). Background ions from the enzyme preparations were determined by spotting samples of enzymes at pH 3 to 10 without NIMS substrates, and by measuring clean areas of the NIMS chip; these background ions were excluded. The peaks were binned into +/- 0.1 m/z bins. Peaks were further binned into “substrate” (mass equivalent to substrate + 1) “modified” (substrate mass − 1 to −70, or + 2 to 68), “cleaved” (substrate mass − 502 to −71), or “polymerized” (substrate mass + 69 to + 1000). Relative proportions of the four bins were calculated by dividing the summed intensity of peaks in a given bin by the summed intensity of peaks in all the bins (Fig. 1c iv). Data was handled with pandas Version 2.2.2^38^. Dunnett analyses (1-sided; enzyme treatments compared to no-enzyme controls) were performed with SciPy Version 1.14.0^39^. In the no-enzyme controls, no signals above background noise were detected in the cleaved category for the 4-O-5’ substrate, nor in the polymerized category for the β-O-4’ substrate, so Dunnett tests were not performed for the 4-O-5’/cleaved or the β-O-4’/polymerized data. Figures were generated with GraphPad Prism (Version 10.2.3 for Windows, GraphPad Software, Boston, Massachusetts USA, www.graphpad.com), BioRender (BioRender.com), ChemDraw (revvitysignals.com), and MatPlotLib Version 3.9.0^40^. Raw MSI data files are viewable at https://metaspace2020.org/project/LigNIMS-2025 with the LigNIMS Database-1.

Overall, we performed over 2,000 reactions to characterize ten enzymes at eight pH conditions, thus taking advantage of the high throughput robotic spotting and NIMS. After a three-hour incubation, it took approximately five minutes to acidify and prepare the samples. Droplet printing took 15 min per 96-well plate. Mass spectrometry imaging took approximately two hours per 96-sample array. The time could be decreased by increasing the laser raster width during imaging, since we obtained an average of 24 pixels per sample spot (Supplementary Figure S3). However, we found that imaging time was not a bottleneck in the throughput of our process and hence we chose a higher resolution in imaging. Using robotic liquid handlers with globally-used commercial pipette tips and 96-well-plates makes the sample transfer method readily transferable to other laboratories. This method of printing coupled with MSI can be highly scalable for any laser desorption-ionization mass spectrometry technique. In comparison to a 12–15 min/sample throughput of a typical gas chromatography mass spectrometry (GC-MS), NIMS required only seconds per sample for acquisition time, and when coupled with printing our approach required approximately 1.5 min/sample for the entire workflow.

## Results and discussion

### Enzyme activity screening

**Fig. 3:**
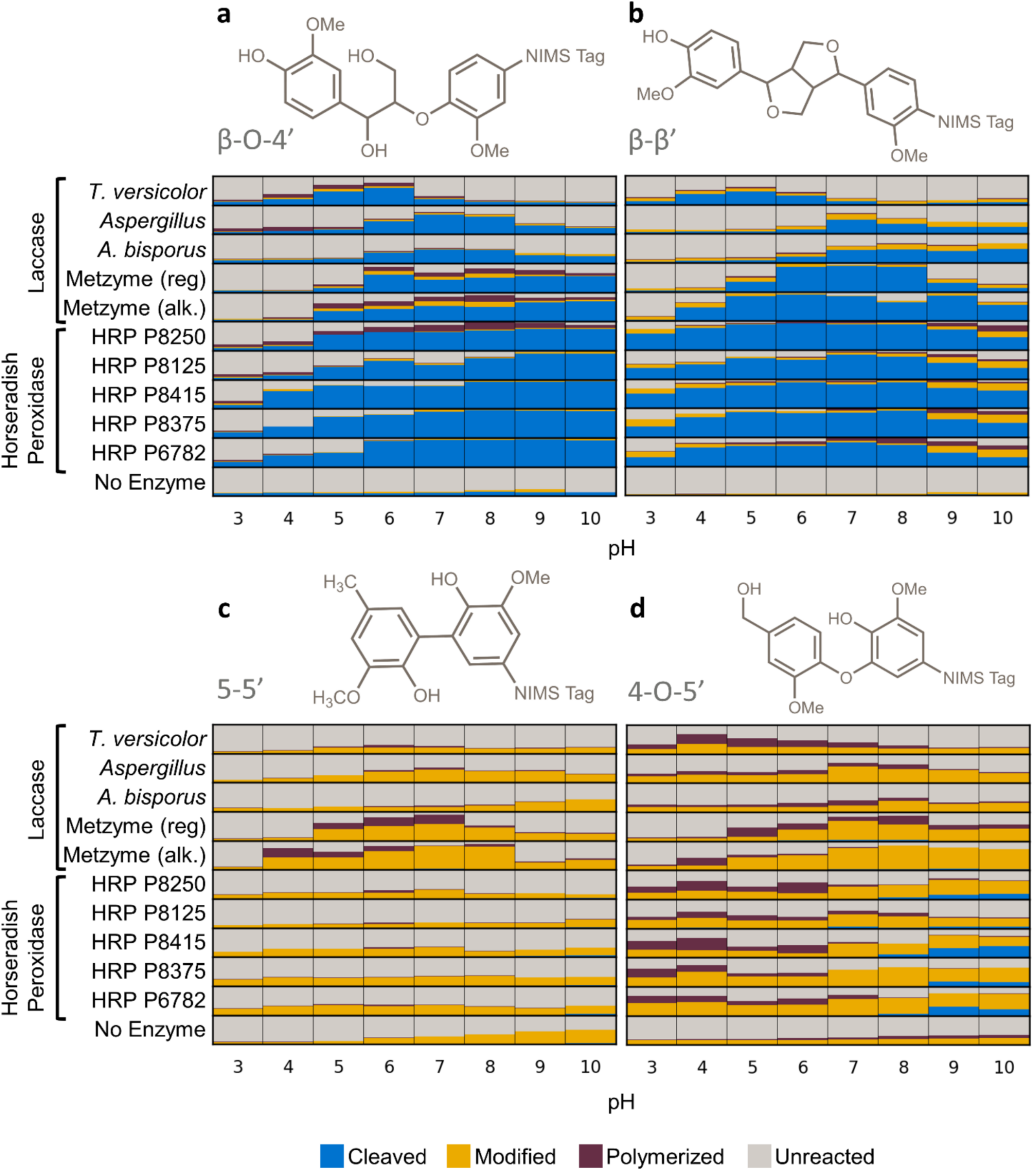
Average relative proportions of products after a 3-hour incubation of 11 different enzymes at pH 3–10 with the **a** β-O-4’ substrate, **b** β-β substrate, **c** 5–5’ substrate, or **d** 4-O-5’ substrate. pH is shown on the x-axis and the enzyme is shown on the y-axis. The size of colored squares represents the relative proportion of ion intensities for unreacted substrate, modified substrate (typically an oxidized product), cleaved substrate, or polymerized substrate. All enzyme reactions with each substrate were repeated in three or four independent repeat experiments. HRP, horseradish peroxidase. Reg., regular Metzyme laccase L111. Alk., alkaliphilic Metzyme laccase L371.

pH is hypothesized to be one of the most important conditions for enzymatic depolymerization of lignin polymers. pH influences the degree of polymerization versus depolymerization by LMEs^24^and developing LMEs that function at pH extremes^12–14 ^would be favorable for the incorporation of LMEs in lignocellulose conversion workflows. Recent studies with NIMS-tagged model lignin substrates representing the β-O-4’ linkage demonstrated that the cleavage of the C-O and C-C bonds in the model compound is a function of pH, presence of mediator (e.g., 1-hydroxybenzotriazole and syringaldehyde) and phenolic vs non-phenolic model compounds^12,25,30,31^. However, little is known about how other bond types, which are abundant in certain types of lignin and pretreated biomass^33,34^ are affected by these factors. To understand how pH and LME type influence the cleavage of different bond types in model lignin dimers, we incubated ten different enzymes (five laccases and five horseradish peroxidases) with four substrates representing the β-O-4’, β-β’, 5–5’, and 4-O-5’ bonds (Figs. 1 and 2). We assessed a range of pH 3 to pH 10. We categorized peaks into the following bins: “unreacted” (substrate mass), “cleaved” (loss of mass of an aromatic ring or more), “modified” (gain or loss of mass less than an aromatic ring), or “polymerized” (gain of mass of an aromatic ring or more). Mass values for modified peaks typically indicated an oxidation event (e.g., addition of 16 *m/z* to the substrate mass). Results of the screen are shown in Fig. 3 and Supplementary Table S1.

All ten enzymes cleaved the β-O-4’ and β-β’ model substrates (Fig. 3a-b), while fewer enzymes cleaved the 5–5’ and 4-O-5’ substrates and to a lesser extent (Fig. 3c-d). The intermediates and products of the β-O-4’ reactions were the same as those previously identified^25^ and resulted from either cleavage of the β-ether bond or C(alpha)-C(aryl) carbon-carbon single bond. The *Aspergillus* sp. laccase and *A. bisporus* laccase cleaved the β-O-4’ and β-β’ model substrates at a higher pH range than the *T. versicolor* laccase. The pH ranges we observed with the NIMS-tagged substrates (Fig. 3; Table 1) aligned with the previous spectrophotometric data that showed trends of higher pH for *A. bisporus* laccase and *Aspergillus* sp. laccase, and lower pH range for *T. versicolor* laccase (Supplementary Table S2^8,35,41,42^). The pH ranges for the Metzyme laccases also correlated with previous data obtained with spectrophotometric assays, with lower pH optima for L111 and higher pH optima for the alkaliphilic L371 (Supplementary Table S2^13^). Both of the Metzyme laccases cleaved the β-O-4’ model substrate at pH 6–10. However, when we tested the β-β’ substrate (Fig. 3b), the pH range for the Metzyme L371 was 4.0–10.0 while the pH range for L111 was 5.0–8.0, suggesting that the active pH range was wider for L371 on the β-β’ compound.

The horseradish peroxidases cleaved both β-β’ and β-O-4’ bond types across a wider pH range than laccases (Fig. 3; Table 1). Our results reflect the previously-reported wide range of optimal pH reported for horseradish peroxidases (Supplementary Table S2^42,43^). The broader pH range for the horseradish peroxidases may be due to the use of H_2_O_2_ as an oxidant, as opposed to laccases, which use O_2_ as an oxidant. Interestingly, the active pH range differed between the β-β’ and β-O-4’ substrates; the horseradish peroxidases modified and cleaved the β-β’ substrate at a lower pH (pH 3 or 4) than the β-O-4’ (generally pH 4 or 5).

**Table 1:** pH activity ranges for NIMS-tagged model lignin compounds determined by maximum total activity (cleaved, modified, and polymerized).

	pH Activity Ranges for NIMS-tagged Substrates			
Enzyme	β-O-4’	β-β’	5–5’	4-O-5’
*T. versicolor* laccase	4–6	4–6	N.D.	4–5
* A. bisporus* laccase	6–8	7–10	N.D.	N.D.
*Aspergillus* sp. laccase	6–8	7–8	N.D.	7–9
Metzyme L111 laccase	6–10	6–8	5–7	6–8
Metzyme L371 laccase	5–10	4–10	4–8	7–10
Horseradish Peroxidases	4 or 5–10	4–10	3–5^a^	3–10^b^

N.D., not determined since activity was either similar to the negative control or highly variable between replicates.

^a^For HRP P8375 and P6782. N.D. for other HRPs.

^b^Variable across different HRPs.

The laccases and horseradish peroxidases also modified the 4-O-5’ and 5–5’ substrates, although potential oxidation products dominated the products and little bond cleavage occurred (Fig. 3c-d). Modification of the 4-O-5’ bond type was much more extensive than the 5–5’ substrate, which is a strong bond. While the horseradish peroxidases appeared to be more active on the β-O-4’ and β-β’ bond types, the Metzyme laccases oxidized a greater percentage of the 5–5’ substrate than the horseradish peroxidases (Fig. 3c). The oxidation by the other laccases was not significantly greater than the no-enzyme control (Supplementary Table S1). These trends may be especially relevant for pre-treated biomass that is enriched in carbon-carbon bonds such as 5–5’ bonds^34^.

We did not observe any relationship between polymerization and pH (Fig. 3) as reported previously^24^. Most conditions did not have much polymerization, which is consistent with our hypothesis that the LigNIMS substrates limit the extent of polymerization^25^. This said we did observe polymerization across more conditions with the 4-O-5’ LigNIMS substrate (Fig. 3d), although these were not the major product (typically less than 40%; Supplementary Table S1). The polymers we observed were typically dimers, with no higher order of oligomers observed.

### Oxidation and bond cleavage pathway of the β-β’ substrate

**Fig. 4:**
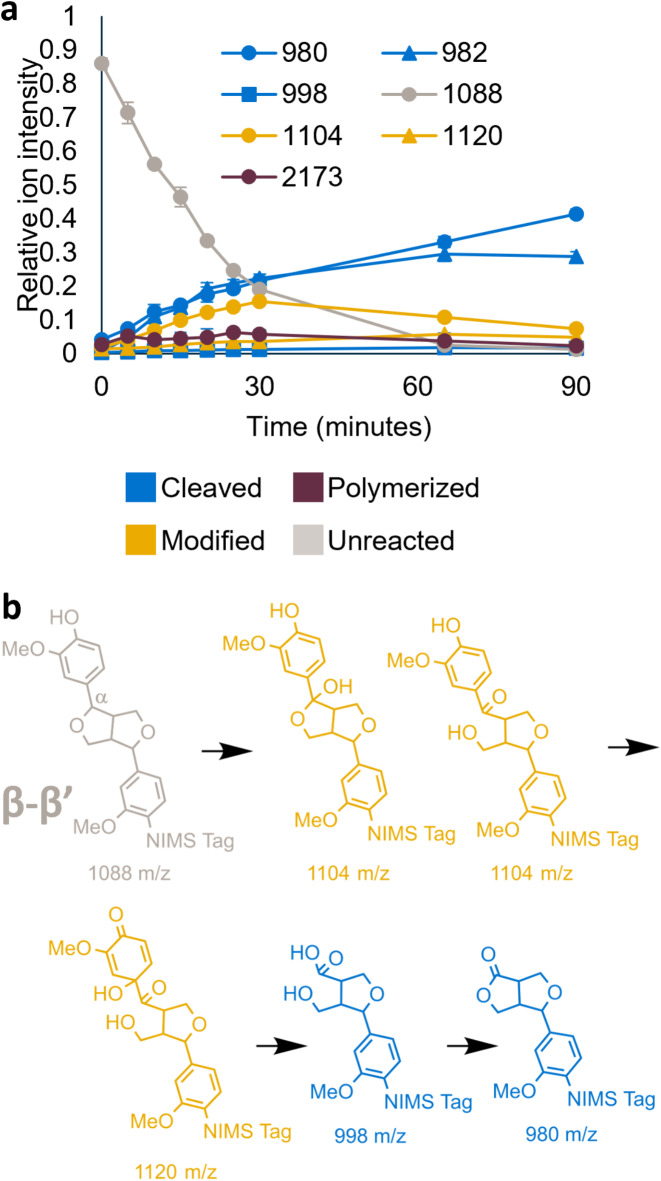
Oxidation and cleavage pathway for the β-β’ substrate. **a** Relative abundance of ions during incubation with *T. versicolor* laccase over a 90-minute incubation at pH 5. Error bars represent standard deviation of two biological replicates. All *m/z* values are shown as [M + H]^+^. **b **Proposed reaction pathway for β-β’ substrate hydroxylation followed by bond cleavage (based on previous studies^23,26^).

All ten laccases and horseradish peroxidases cleaved the β-β’ substrate (Fig. 3b). We further evaluated commercially available laccase from *T. versicolor* with the β-β substrate at pH 5 (the pH with the highest activity in the screen) and at room temperature. The mass spectra indicated that the laccase induces bond cleavage, with sequential increase in cleavage products and intermediate production of an oxidized product with a mass of 1104 *m/z* (Fig. 4a). Figure 4b shows a proposed pathway for NIMS-tagged β-β’ substrate oxidation and subsequent cleavage of the carbon-carbon bond between the aromatic ring and the ɑ carbon. The pathway is based on previously published pathways for syringaresinol cleavage by fungus *Fusarium solani *M-13-1^23^ and pinoresinol cleavage by *Pseudomonas *sp. strain SG-MS2^26^. Both microorganisms oxidized the resinol substrates and subsequently cleaved the ɑ-aryl bond. We detected ions with *m/z* values of 1104, 1120, 998, and 980, which support the proposed pathway with oxidation (1104 and 1120 *m/z* ions) and cleavage (998 and 980 *m/z* ions). Additionally, we detected 982 *m/z* peaks, which may be a further oxidized version of the 980 *m/z* product. Most products were the result of cleavage of the C(alpha)-C(aryl) carbon-carbon bond; however, small peaks were sometimes observed at 898 m/z, suggesting possible cleavage of other bonds. We also detected small 2173 *m/z* peaks, which are likely dimers of the substrate.

The pH of the reaction affected the relative cleaved product distribution (Supplementary Figure S4). The major cleavage products had *m/z* values of 998, 980, and 982. The structures for the 998 and 980 *m/z* peaks are hypothesized based on previous publications (Fig. 4b^23,26^), while the structure of the 982 *m/z* peak is unknown. When examining the relative proportion of these three putative products, we calculated a negative correlation (*P* < 0.0001) between pH and the 980 *m/z* product, and a positive correlation (*P* < 0.0001) between pH and the 998 *m/z* product (Supplementary Figure S4). Basic conditions favor the hydrolysis of lactone compounds to the open chain hydroxycarboxylic acid, and in our study, we noted that the hydrolyzed product (998 *m/z*) is favored over the lactone product (980 *m/z*) as pH increases, which reflect the equilibrium dynamics between these two products by pH change.

It was hypothesized that the syringyl form of aromatic linkages are more prone to bond cleavage, whereas the oxidation of guaiacol-type aromatic linkages leads to polymerization^24^. Past laccase research demonstrated that laccase cleaves syringaresinol^23 ^but laccase and peroxidase polymerize pinoresinol^21^, contrary to the cleavage we observed with the NIMS-tagged pinoresinol in our study. This further demonstrates that the NIMS tag restricts polymerization^25^ and enables the observation of resinol dimer cleavage mechanisms without the complication of polymerization of substrates and condensation of products.

### Oxidation of the 4-O-5’ and 5–5’ substrates

Most enzymes oxidized the 4-O-5’ substrate and the Metzyme laccases and several peroxidases oxidized the 5–5’ bonds (Fig. 3c-d), but little to no bond cleavage occurred for these two model substrate types. They are in the syringyl form; that is, the 5’ carbon located ortho to the phenolic group is occupied (Fig. 1b^24^). When the 5’ carbon is unoccupied in untagged model compounds, it is highly reactive and leads to polymerization^24,44^. However, since our NIMS-tag limits polymerization, it may be that the guaiacol-type substrates (with unoccupied 5’ carbons) are more prone to radical formation and subsequent bond cleavage.

Common products for the 5–5’ model substrate were 978 *m/z* ([M + 16]) and 994 *m/z *([M + 32]). These are likely oxidation products with the addition of oxygen molecules, similar to the first step of oxidation of β-O-4’ and β-β’ substrates^22,23^. Similarly, common modified products of the 4-O-5’ substrate were 994 ([M + 16]) and 1010 *m/z* ([M + 32]), which are likely from the addition of one and two oxygen atoms, respectively. We also noted a 960 *m/z* product for the 5–5’ substrate and 976 *m/z*product for the 4-O-5’ substrate ([M-2]), which may be oxidation of the primary alcohols as previously seen with the oxidation of the β-O-4’ substrate^25^. Surprisingly, the 4-O-5’ reactions also had a high abundance of a product at 962 *m/z* ([M-16]), suggesting a loss of an oxygen atom. Further research is needed to determine the exact structure of products from the 4-O-5’ and 5–5’ reactions.

Past experiments demonstrated that a mediator is often required for laccases to oxidize non-phenolic substrates^8,36^. While all the NIMS-tagged substrates in this study were phenolic, we hypothesized that the inclusion of mediator would enable the cleavage of 5–5’ and 4-O-5’ bonds, where we observed oxidation but little to no bond cleavage (Fig. 3c-d). We tested three of the laccases (L111, L371, and *Aspergillus* sp. laccase) with and without 1-hydroxybenzotriazole as a mediator. There were no significant changes in the degree of polymerization, modification, or cleavage for either substrate (Supplementary Figures S5 and S6).

### Limitations and future work

We reported relative abundances of ions (i.e., ratios of ion intensities to total ion intensity) which is not fully quantitative. Another limitation of NIMS is that the NIMS chip is not commercially available and must be prepared in house following previously published protocols^45^ and the substrates must be synthesized (see Supplemental Methods). With the detailed bond-breaking data that NIMS provides and the high-throughput platform we developed, we find that the value justifies the labor.

As with other model lignin compounds (both dimers and monomers), NIMS-tagged dimers do not fully mimic the reactivity of native lignin. We suggest that NIMS-tagged lignin dimers provide a rapid method for screening the activity of LMEs, and that the micelle structure of the NIMS substrates prevents the polymerization reactions that often complicate chemical analysis. While reactions with NIMS-tagged dimers may approximate what happens to a bond initially, we do not observe potential repolymerization reactions that are likely to occur with native lignin. Understanding what conditions influence initial bond cleavage is useful to the lignin valorization industry, but research into the factors that influence repolymerization of cleavage products is crucial as well. We recognize that continued investment into improving native lignin analytics is needed and ultimately activity of LMEs on model substrates will need substantiation on native lignin. Future work could include testing LMEs on native lignin at different pH and determining if the pH profiles and bond-specificity observed in our study holds true with native lignin; however, such experiments are difficult with current lignin analytical methods.

## Conclusions

The goal of this study was to determine the activity and pH range of LMEs with surrogate substrates representing the different bond types found in lignin in a setting that limits polymerization. We tested a set of four NIMS-tagged model lignin dimers to rapidly screen LME activity on the β-O-4’, β-β’, 5–5’, and 4-O-5’ lignin bond types under a range of pH conditions. Previous studies typically focused on monomers or dimers with the β-O-4’ bond, which are limited by the propensity of such compounds to polymerize. Our approach allowed detailed examination of bond cleavage mechanisms for multiple types of bonds without laborious chromatography or NMR methodologies. We demonstrated that laccases and horseradish peroxidases can cleave β-O-4’ and β-β’ model dimers and oxidize 5–5’ and 4-O-5’ model dimers when the model substrates are NIMS-tagged. Although previously we knew LMEs such as laccases could cleave β-O-4’^22 ^and β-β’ bonds^21^, very few studies report how LMEs might cleave 5–5’ or 4-O-5’ bond types and characterization of LMEs at a range of pH on all four bond types were limited. Our data suggest that the (a) enzyme type, (b) pH, and (c) bond type determine the extent of oxidation versus substrate cleavage. Interestingly, the active pH range for a given enzyme varied between different bond types. The relative abundance of different bond types in lignin (which vary by feedstock and pretreatment method) may therefore play an important role when choosing the enzyme and process conditions for biological lignin depolymerization.

## Electronic supplementary material

Below is the link to the electronic supplementary material.

Supplementary Material 1

Supplementary Material 2

## Data Availability

The datasets supporting the conclusions of this article are available within the article, at Metaspace (https://metaspace2020.org/project/LigNIMS-2025), and within the article’s supplementary files.

## Abbreviations

ABTS: 2,2′-Azino-bis(3-ethylbenzthiazoline-6-sulfonic acid)
DMSO: Dimethyl sulfoxide
GC-MS: Gas chromatography-mass spectrometry
G-O-4’: Guaiacyl-glycerol-β-guaiacyl ether
HRP: Horseradish peroxidase
LMEs: Lignin-modifying enzymes
MALDI-TOF-MS: Matrix-assisted laser desorption/ionization time-of-flight mass spectrometry
MSI: Mass spectrometry imaging
NIMS: Nanostructure-initiator mass spectrometry
NMR: Nuclear magnetic resonance
OMAAT: OpenMSI Arrayed Analysis Toolkit
S-O-4’: Syringyl-glycerol-β-guaiacyl ether
TFA: Trifluoroacetic acid
