# Supplementary material for: Enzymatic cleavage of model lignin dimers depends on pH, enzyme, and bond type
Source: Sci Rep. 2025 Mar 25;15:10296. doi: 10.1038/s41598-025-88571-7 (PMC11937299; doi:10.1038/s41598-025-88571-7)
Supplement: Supplementary file 2 — Supplementary Material 2 [file 41598_2025_88571_MOESM2_ESM.docx]

**Supplementary Information**

**Enzymatic cleavage of model lignin dimers depends on pH, enzyme, and bond type**

Jenny R. Onley^1,2^, Kshitiz Gupta^1,3^, Markus de Raad^4^, Benjamin P. Bowen^4^, Stephen Tan^1,5^, Sam Yoder^1,5^, Kenneth L. Sale^6,7^, Anup K. Singh^1,3^, Blake A. Simmons^5,7^, Paul D. Adams^1,4^, Trent R. Northen^1,4^, Kai Deng^1,2*^

^1^Technology Division, Joint BioEnergy Institute, Emeryville, CA, USA

^2^Biomaterials and Biomanufacturing Department, Sandia National Laboratories, Livermore, California, USA

^3^Engineering Directorate, Lawrence Livermore National Laboratory, Livermore, California, USA

^4^Environmental Genomics and Systems Biology Division, Lawrence Berkeley National Laboratory, Berkeley, CA, USA

^5^Biological Systems and Engineering Division, Lawrence Berkeley National Laboratory, Berkeley, CA, USA

^6^Biosecurity and Bioassurance Department, Sandia National Laboratories, Livermore, California, USA

^7^Deconstruction Division, Joint BioEnergy Institute, Emeryville, CA, USA

*Correspondence: Kai Deng (kdeng@sandia.gov)

Contents:

1. Supplementary Methods
2. Supplementary Figures

**1. Supplementary Methods**

***General Information about Organic Synthesis***

All chemicals were purchased as reagent grade and used without further purification. Flash column chromatography steps were performed on a CombiFlash NextGen 300 chromatography system from Teledyne ISCO (Lincoln, NE). Reactions were monitored using analytical thin-layer chromatography (TLC) in EM silica gel 60 F254 plates and developed by acidic ceric ammonium molybdate or potassium permanganate TLC stains. ^1^H NMR and ^13^C NMR spectra were recorded on a Bruker AV-600. Chemical shifts (in ppm) were assigned according to the internal standard signal of CDCl_3_ (δ = 7.26 ppm), CD_3_OD (δ = 3.31 ppm), or CDCl_3_ (δ = 77.16 ppm) and CD_3_OD (δ = 49.00 ppm) for ^13^C NMR. Coupling constants (J) are reported in Hertz, and the splitting patterns are described by using the following abbreviations: s, singlet; d, doublet; dd, doublet of doublets; ddd, doublet of doublet of doublets; t, triplet; dt, doublet of triplets; q, quartet; m, multiple; AB, AB spin system. High-resolution mass spectral data were obtained from the University of California, Berkeley Mass Spectral Facility.


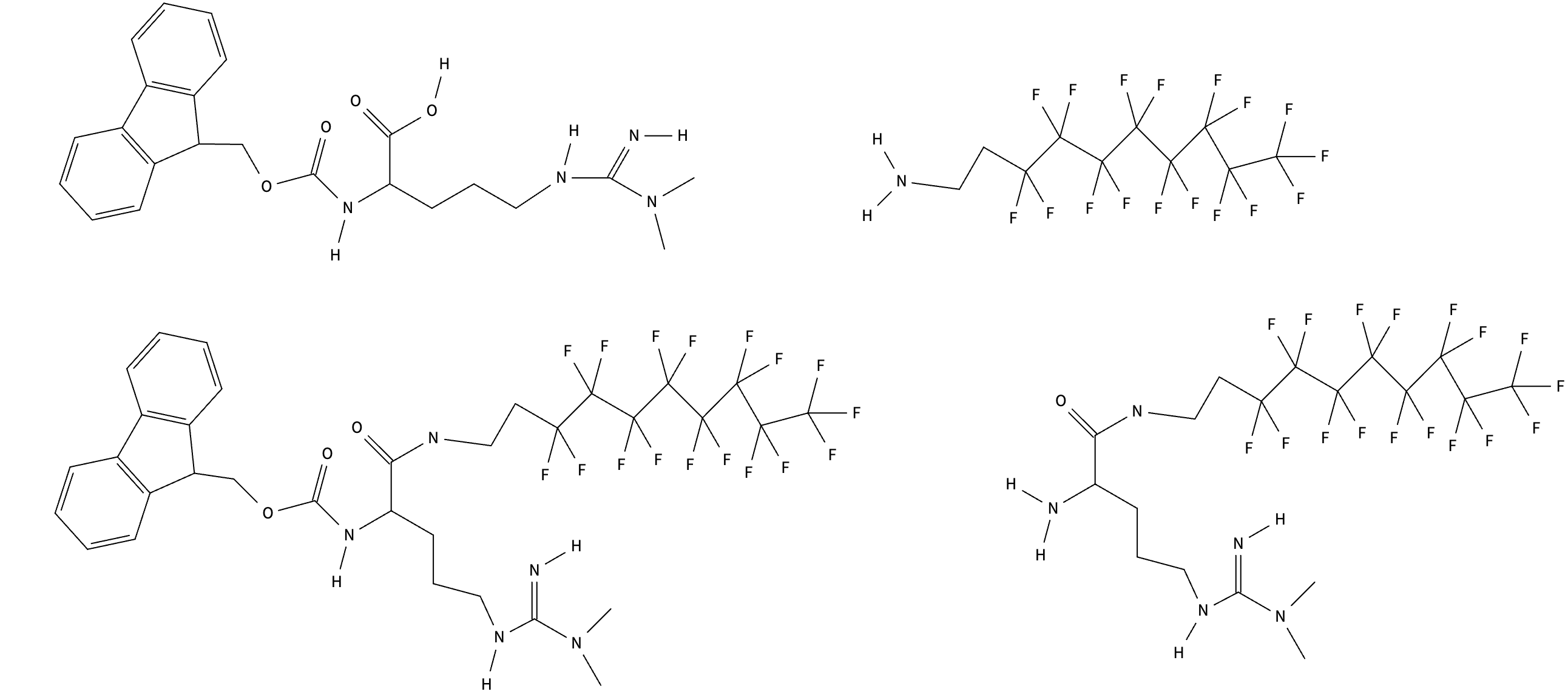


DEN_E_174

Piperidine

DMF

DEN_E_175

PyBOP, DIPEA, DMF

+

**E174**

To a stirred solution of Fmoc-Arg(Me)_2_-OH (110 mg, 0.26 mmol) and NH_2_CH_2_CH_2_C_8_F_17_ (144 mg, 0.31 mmol) in anhydrous DMF (2.5 ml) at room temperature was added PyBOP (161 mg, 0.31 mmol), followed by the addition of DIPEA (0.136 mL, 0.78 mmol). The resulting mixture was stirred for 2 h. After concentration under reduced pressure, the residue was purified by column chromatography to give 160 mg product E174 in 71% yield. ^1^H NMR (600 MHz, CDCl_3_) δ (ppm) 7.79 (d, 2H, J = 9.0 Hz), 7.66 (t, 2H, J = 9.6 Hz), 7.38 (t, 2H, J = 8.8 Hz), 7.30 (t, 2H, J = 8.8 Hz), 4.44-4.38 (m, 2H), 4.22 (t, 1H, J = 7.9 Hz), 4.12-4.05 (m, 1H), 3.55-3.49 (t, 2H, J = 8.2 Hz), 3.27-3.22 (m, 2H), 3.00 (s, 6H), 2.50-2.35 (m, 2H), 1.88-1.80 (m, 1H), 1.72-1.60 (m, 3H). NMR (600 MHz, CD_3_OD) δ (ppm) 3.54 (t, 2H, J = 8.5Hz), 3.36 (t, 1H, J = 8.4 Hz), 3.24 (t, 2H, J = 8.2 Hz), 3.03 (s, 6H), 2.52-2.36 (m, 2H), 1.85-1.58 (m, 4H). ^13^C NMR (150 MHz, CD_3_OD) 173.33, 157.15, 156.29, 143.93, 143.74, 141.24, 141.22, 127.42 (2C), 126.78 (2C), 124.79, 124.74, 119.57 (2C), 66.58, 54.63, 54.46, 41.47, 37.09 (2C), 31.24, 29.85, 28.71, 25.00.

**E175**

To a stirred solution of E174 (580 mg, 0.67 mmol) in anhydrous DMF (10 ml) at room temperature was added piperidine (0.1 mL). The resulting mixture was stirred for 1 h. After concentration under reduced pressure, the residue was purified by column chromatography to give 370 mg product E175 in 86% yield. ^1^H NMR (600 MHz, CD_3_OD) δ (ppm) 3.54 (t, 2H, J = 8.5Hz), 3.36 (t, 1H, J = 8.4 Hz), 3.24 (t, 2H, J = 8.2 Hz), 3.03 (s, 6H), 2.52-2.36 (m, 2H), 1.85-1.58 (m, 4H). ^13^C NMR (150 MHz, CD_3_OD) 175.68, 156.32, 54.09, 44.39, 41.17, 37.08 (2C), 31.24, 24.80, 22.40.

E178

E177

**E26**

E004 and E006 are known compounds and are prepared according to the literature^[[1]](#endnote-1)^. To a stirred solution of E006 (1.30 g, 3.37 mmol) in a mixture of anhydrous ethanol (45 ml) and anhydrous dichloromethane (15 mL) at -20 ℃ was added sodium borohydride (120 mg, 3.17 mmol). The resulting mixture was stirred for 30 min before the addition of a water/diethyl ether mixture. The aqueous layer was extracted by diethyl ether three times and the combined ethers were dried by MgSO_4_. After filtration and concentration under reduced pressure, the residue was purified by column chromatography to give 1.15 g product E26 in 88% yield.

^1^H NMR (600 MHz, CDCl_3_) δ (ppm) 7.02 (s, 1H), 7.01 (s, 1H), 6.78 (s, 1H), 6.77 (s, 1H), 4.62 (s, 4H), 3.84 (s, 6H), 2.09 (s, 6H). ^13^C NMR (150 MHz, CDCl_3_) 168.89, 151.35, 139.34, 136.77, 131.31, 120.79, 110.46, 64.77, 56.09, 20.46.

**E27C1**

To a stirred solution of E26 (510 mg, 1.31 mmol) in anhydrous dichloromethane (30 mL) at 0 ℃ was added imidazole (133 mg, 1.95 mmol) and TBSCl (225 mg, 1.49 mmol). The resulting mixture was allowed to rise to room temperature and stirred for 12 h. After concentration under reduced pressure, the residue was purified by column chromatography to give 386 mg product E27C1 in 58% yield. 51 mg of E26 was also recovered. ^1^H NMR (600 MHz, CDCl_3_) δ (ppm) 6.92 (s, 1H), 6.89 (s, 1H), 6.66 (s, 1H), 6.64 (s, 1H), 4.61 (s, 2H), 4.44 (s, 2H), 3.72 (s, 3H), 3.67 (s, 3H), 1.97 (s, 3H), 1.95 (s, 3H), 0.94 (s, 9H), 0.11 (s, 6H). ^13^C NMR (150 MHz, CDCl_3_) 169.07, 168.87, 151.28, 151.17, 139.68, 139.56, 136.57, 136.23, 131.32, 131.04, 120.65, 119.76, 110.35, 109.68, 64.54, 64.36, 55.96, 55.92, 25.95, 20.42, 20.39, 18.39, -5.22.

**E39**

To a stirred solution of E27C1 (356 mg, 0.706 mmol) in anhydrous dichloromethane (10 mL) at room temperature was added Dess Martin Periodinane (360 mg, 0.85 mmol). The resulting mixture was stirred for 45 min. After filtration and concentration under reduced pressure, the residue was purified by column chromatography to give product E39 (312 mg, 0.62 mmol) in 88% yield.

^1^H NMR (600 MHz, CDCl_3_) δ (ppm) 9.92 (s, 1H), 7.51 (d, 1H, *J* = 1.8 Hz), 7.37 (d, 1H, J = 1.8 Hz), 7.07 (d, 1H, J = 1.8 Hz), 6.77 (m, 1H), 4.73 (s, 2H), 3.92 (s, 3H), 3.86 (s, 3H), 2.12 (s, 3H), 2.08 (s, 3H), 0.95 (s, 9H), 0.11 (s, 6H). ^13^C NMR (150 MHz, CDCl_3_) 191.01, 168.64, 167.91, 152.30, 151.32, 142.89, 140.06, 136.29, 134.45, 132.58, 129.91, 127.21, 119.42, 110.15, 109.49, 64.46, 56.29, 56.02, 25.93, 20.41, 20.39, 18.40, -5.25.

**E40**

To a stirred solution of E39 (300 mg, 0.597 mmol) in anhydrous dichloromethane (10 mL) at 0 ℃ was added Ph_3_P=CHCO_2_*t*Bu (450 mg, 1.19 mmol). The resulting mixture was allowed to rise to room temperature and stirred for 12 h. After concentration under reduced pressure, the residue was purified by column chromatography to give product E40 (320 mg, 0.53 mmol) in 89% yield.

^1^H NMR (600 MHz, CDCl_3_) δ (ppm) 7.52 (d, 1H, *J* = 16.0 Hz), 7.09 (d, 1H, J = 1.6 Hz), 7.05 (d, 1H, J = 1.2 Hz), 7.00 (d, 1H, J = 1.6 Hz), 6.74 (s, 1H), 7.31 (d, 1H, *J* = 16.0 Hz),

4.72 (s, 2H), 3.88 (s, 3H), 3.86 (s, 3H), 2.09 (s, 3H), 2.08 (s, 3H), 1.53 (s, 9H), 0.95 (s, 9H), 0.10 (s, 6H). ^13^C NMR (150 MHz, CDCl_3_) 168.65, 168.37, 166.09, 151.60, 151.28, 142.72, 139.83, 139.10, 136.28, 132.82, 132.16, 130.50, 123.12, 120.63, 119.57, 110.28, 109.90, 80.61, 64.51, 56.10, 55.99, 28.20, 25.95, 20.43, 20.42, 18.41, -5.33.

**E41**

To a stirred solution of E40 (340 mg, 0.567 mmol) in methanol (15 mL) at room temperature was added 10% Pd/C (Degussa type, 50% wet) (60 mg, 0.0283 mmol). Hydrogen balloon was used to maintain 1 atm H_2_ for 12 h. After filtration and concentration under reduced pressure, the residue was purified by column chromatography to give product E39 (240 mg, 0.508 mmol) in 90% yield.

^1^H NMR (600 MHz, CDCl_3_) δ (ppm) 6.81 (d, 1H, J = 1.9 Hz), 6.77 (d, 1H, J = 1.7Hz), 6.67 (d, 1H, J = 1.8 Hz), 6.63 (d, 1H, J = 1.8, 0.6 Hz), 3.82 (s, 6H), 2.89 (t, 2H, J = 7.8 Hz), 2.54 (t, 2H, J = 8.4 Hz), 2.33 (s, 3H), 2.09 (s, 3H), 2.08 (s, 3H), 1.42 (s, 9H). ^13^C NMR (150 MHz, CDCl_3_) 172.13, 168.81 (2C), 151.04, 150.90, 138.88, 135.96, 135.88, 135.31, 131.45, 131.13, 123.02, 122.32, 112.73, 111.98, 80.46, 56.03, 56.00, 53.43, 36.98, 31.01, 28.08, 21.43, 20.46.

**E94**

To a stirred solution of E41 (144 mg, 0.31 mmol) in dichloromethane (2 mL) at room temperature was added trifluoroacetic acid (0.4 mL). The resulting mixture was stirred for 16 h. After concentration under reduced pressure, the residue was purified by column chromatography to give product E94 (120 mg, 0.288 mmol) in 93% yield. ^1^H NMR (600 MHz, CDCl_3_) δ (ppm) 6.82 (d, 1H, J = 2.0 Hz), 6.78 (d, 1H, J = 1.8 Hz), 6.69 (d, 1H, J = 1.8 Hz), 6.64 (d, 1H, J = 1.8, 0.6 Hz), 3.84 (s, 3H), 3.83 (s, 3H), 2.95 (t, 2H, J = 7.6 Hz), 2.70 (t, 2H, J = 7.6Hz), 2.34 (s, 3H), 2.10 (s, 3H), 2.09 (s, 3H). ^13^C NMR (150 MHz, CDCl_3_) 177.83, 169.36, 169.16, 151.14, 150.86, 138.22, 136.16, 136.01, 135.21, 131.56, 130.98, 122.95, 122.26, 112.80, 111.96, 56.05, 56.01, 35.33, 30.51, 21.44, 20.47, 20.44.

**E177**

To a stirred solution of E94 (81 mg, 0.195 mmol) and E175 (137 mg, 0.20 mmol) in anhydrous DMF (5 ml) at room temperature was added PyBOP (125 mg, 0.24 mmol), followed by the addition of DIPEA (0.11mL, 0.60 mmol). The resulting mixture was stirred for 12h. After concentration under reduced pressure, the residue was purified by column chromatography to give 154 mg product E177 in 76% yield. ^1^H NMR (600 MHz, CDCl_3_) δ (ppm) 6.95 (t, 1H, J = 5.8 Hz), 6.87 (d, 1H, J = 1.6 Hz), 6.77 (d, 1H, J = 1.2 Hz), 6.70 (d, 1H, J = 7.8 Hz), 6.64 (br, 1H), 6.61 (br, 1H), 6.08 (br, 2H), 4.38-4.31 (m, 1H), 3.809 (s, 3H), 3.806 (s, 3H), 3.52-3.43 (m, 2H), 3.26-3.12 (m, 2H), 2.96 (s, 6H), 2.93-2.85 (m, 2H), 2.62-2.56 (t, 2H, J = 7.2 Hz), 2.32 (s, 3H), 2.36-2.26 (m, 2H), 2.08 (s, 3H), 1.79-1.68 (m, 1H), 1.65-1.54 (m, 1H), 1.54-1.45 (m, 1H), 1.45-1.36 (m, 1H). ^13^C NMR (150 MHz, CDCl_3_) 173.27, 172.17, 169.51, 169.49, 155.74, 151.15, 150.89, 138.82, 136.27, 135.79, 135.21, 131.21, 130.83, 122.86, 122.19, 112.78, 112.17, 56.05, 55.95, 52.38, 42.02, 37.84, 37.28, 31.88, 31.19, 30.38, 30.24, 30.09, 24.34, 21.26, 20.42, 20.38.

**E178**

To a stirred solution of E177 (50 mg, 0.048 mmol) methanol (10 ml) at room temperature was added hydrazine (200ul). The resulting mixture was stirred for 3h. After concentration under reduced pressure, the residue was purified by column chromatography to give 40 mg product E178 in 87% yield. ^1^H NMR (600 MHz, CD_3_OD) δ (ppm) 6.81 (d, 1H, J = 1.8 Hz), 6.80 (d, 1H, J = 1.2 Hz), 6.67 (d, 1H, J = 1.8 Hz), 6.64 (d, 1H, J = 1.8 Hz), 4.13 (dd, 1H, J = 9.6, 4.2 Hz), 3.791 (s, 3H), 3.788 (s, 3H), 3.44 (t, 2H, J = 7.2 Hz), 2.92 (t, 2H, J = 7.8 Hz), 2.89 (s, 6H), 2.87 (t, 2H, J = 7.2 Hz), 2.54 (m, 2H), 2.40-2.30 (m, 2H), 2.28 (s, 3H), 1.61-1.53 (m, 1H), 1.35-1.27 (m, 1H), 1.06-0.93 (m, 2H). ^13^C NMR (150 MHz, CD_3_OD) 174.40, 172.96, 156.11, 150.55, 150.11, 148.16, 146.09, 129.14, 129.05, 127.64, 125.83, 122.56, 122.54, 110.51, 110.18, 54.94, 54.85, 52.64, 48.45, 41.41, 37.84, 37.06 (2C), 31.37, 28.54, 24.63, 19.95 (2C).

**E184**

**E49C1**^[[2]](#endnote-2)^

To a stirred solution of vinily alcohol (400 mg, 2.56 mmol) in anhydrous acetone (10 mL) was added Ag_2_(I)O (700 mg, 3.02 mmol). The resulting mixture was stirred at room temperature for 0.5 h. After filtration and concentration under reduced pressure, the residue was purified by column chromatography to give 140 mg product E49C1 in 18% yield. ^1^H NMR (600 MHz, CD_3_OD) δ (ppm) 9.64 (S, 1H), 7.22 (d, 1H, *J* = 1.6 Hz), 7.09 (d, 1H, J = 1.6 Hz), 6.99-6.89 (m, 3H), 4.64 (s, 2H), 3.99 (s, 3H), 3.86 (s, 3H). ^13^C NMR (150 MHz, CD_3_OD) 191.37, 150.81, 148.98, 145.56, 143.70, 143.35, 138.62, 127.63, 120.17, 119.49, 113.79, 111.67, 106.48, 64.03, 66.23, 55.82.

**E65**

To a stirred solution of E49C1 (120 mg, 0.394 mmol) in anhydrous dichloromethane (10 mL) at 0 ℃ was added Ph_3_P=CHCO_2_Me (260 mg, 0.78 mmol). The resulting mixture was allowed to rise to room temperature and stirred for 12 h. After concentration under reduced pressure, the residue was purified by column chromatography to give product E65 (120 mg, 0.33 mmol) in 85% yield. ^1^H NMR (600 MHz, CD_3_OD) δ (ppm) 7.46 (d, 1H, *J* = 15.6 Hz), 7.13 (d, 1H, J = 1.2 Hz), 6.98 (s, 1H), 6.94-6.86 (m, 2H), 6.54 (d, 1H, *J* = 1.8 Hz), 6.20 (dd, 1H, J = 15.6, 0.6 Hz), 4.60 (s, 2H), 3.92 (s, 3H), 3.83 (s, 3H), 3.72 (s, 3H). ^13^C NMR (150 MHz, CD_3_OD) 168.0, 150.88, 149.04, 145.61, 145.01, 144.29, 139.59, 138.37, 125.11, 119.57, 119.24, 114.53, 111.74, 110.63, 106.17, 63.56, 55.52, 55.08, 50.62.

**E133**

To a stirred solution of E65 (50 mg, 0.139 mmol) in methanol (8 mL) at room temperature was added 10% Pd/C (Degussa type, 50% wet) (15 mg, 0.007 mmol). Hydrogen balloon was used to maintain 1 atm H_2_ for 12 h. After filtration and concentration under reduced pressure, the residue was purified by column chromatography to give product E133 (48 mg, 0.132 mmol) in 95% yield.

^1^H NMR (600 MHz, CD_3_OD) δ (ppm) 7.09 (d, 1H, *J* = 1.8 Hz), 6.87 (dd, 1H, J = 7.8, 1.8 Hz), 6.80 (d, 1H, J = 8.4 Hz), 6.58 (d, 1H, *J* = 9.0 Hz), 4.57 (s, 2H), 3.85 (s, 3H), 3.83 (s, 3H), 3.57 (s, 3H), 2.73 (t, 2H, J = 7.8 Hz), 2.50 (t, 2H, J = 7.8 Hz). ^13^C NMR (150 MHz, CD_3_OD) 173.71, 150.69, 148.76, 145.00, 144.99, 137.68, 135.29, 131.31, 119.17, 118.99, 111.69, 110.26, 106.76, 63.58, 55.37, 55.11, 50.65, 35.47, 30.34.

**E154**

To a stirred solution of E133 (60 mg, 0.166 mmol) in a mixture of methanol (3 mL) and water (2mL) was added 0.5 M NaOH (1 mL). The resulting mixture was stirred at room temperature for 12 h. After concentration under reduced pressure, the residue was purified by column chromatography to give product E154 (48 mg, 0.138 mmol) in 83% yield. ^1^H NMR (600 MHz, CD_3_OD) δ (ppm) 7.09 (d, 1H, *J* = 2.0 Hz), 6.86 (dd, 1H, J = 8.0, 2.0 Hz), 6.80 (d, 1H, J = 8.0 Hz), 6.60 (d, 1H, *J* = 2.5 Hz), 6.22 (d, 1H, J = 2.0 Hz), 4.57 (s, 2H), 3.85 (s, 3H), 3.83 (s, 3H), 2.72 (t, 2H, J = 10.0 Hz), 2.47 (t, 2H, J = 10.0 Hz). ^13^C NMR (150 MHz, CD_3_OD) 175.34, 150.61, 148.74, 145.02, 144.88, 137.53, 135.24, 131.58, 119.22, 118.83, 111.66, 110.37, 106.79, 63.62, 55.36, 55.11, 35.55, 30.39.

**E184**

To a stirred solution of E154 (23 mg, 0.064 mmol) and E175 (34 mg, 0.053 mmol) in anhydrous DMF (1.5 ml) at room temperature was added PyBOP (34 mg, 0.064 mmol), followed by the addition of DIPEA (28µL, 0.16 mmol). The resulting mixture was stirred for 12h. After concentration under reduced pressure, the residue was purified by column chromatography to give 40 mg product E184 in 77% yield.

^1^H NMR (600 MHz, CD_3_OD) δ (ppm) 7.10 (d, 1H, *J* = 1.8 Hz), 6.86 (dd, 1H, J = 8.4, 1.8 Hz), 6.76 (d, 1H, J = 7.8 Hz), 6.62 (d, 1H, *J* = 1.8 Hz), 6.27 (d, 1H, J = 1.8 Hz), 4.57 (s, 2H), 4.24 (dd, 1H, J = 9.0, 5.4 Hz), 3.87 (s, 3H), 3.85 (s, 3H), 3.51-3.47 (m, 2H), 3.23-3.15 (m, 2H), 3.03 (s, 6H), 2.80-2.75 (m, 2H), 2.51-2.46 (m, 2H), 2.45-2.35 (m, 2H), 1.80-1.74 (m, 1H), 1.58-1.51 (m, 1H), 1.50-1.38 (m, 2H). ^13^C NMR (150 MHz, CD_3_OD) 173.94, 172.71, 156.26, 150.36, 148.84, 145.17, 144.57, 137.44, 135.57, 131.38, 119.15, 118.31, 111.56, 111.08, 107.24, 63.52, 63.03, 55.42, 55.09, 52.62, 48.16, 41.52, 37.28, 37.15 (2C), 30.99, 28.60, 24.87.

**E188**

**E180**

To a stirred solution of (-)-Pinoresinol (85 mg, 0.237 mmol) in DMF (6 mL) at room temperature was added potassium carbonate (66 mg, 0.48 mmol) followed by the dropwise addition of methyl-5-bromovalerate (31 mg, 0.16 mmol) The resulting mixture was stirred for 12 h. After filtration and concentration under reduced pressure, the residue was purified by column chromatography to give product E180 (42 mg, 0.089 mmol) in 56% yield. ^1^H NMR (600 MHz, CDCl_3_) δ (ppm) 6.92-6.88 (m, 3H), 6.86-6.81 (m, 3H), 5.61 (s, 1H), 4.75 (d, 1H, J = 4.5 Hz), 4.74 (d, 1H, J = 4.5 Hz), 4.28-4.22 (m, 2H), 4.02 (t, 2H, J = 6.3 Hz), 3.91 (s, 3H), 3.90-3.88 (m, 2H), 3.87 (s, 3H), 3.67 (s, 3H), 3.14-3.07 (m, 2H), 2.40 (t, 2H, J = 7.4 Hz), 1.90-1.79 (m, 4H). ^13^C NMR (150 MHz, CDCl_3_) 173.91, 149.71, 148.05, 146.72, 145.26, 133.79, 132.95, 118.98, 118.30, 114.28, 113.04, 109.85, 108.62, 85.91, 85.79, 71.74, 71.71, 68.61, 56.08, 55.97, 54.20, 54.15, 51.54, 33.70, 28.64, 21.57.

**E182**

To a stirred solution of E180 (40 mg, 0.085 mmol) in a mixture of methanol (1.2 mL) and water (0.8 mL) was added 0.5 M NaOH (0.6 mL). The resulting mixture was stirred at room temperature for 12 h. After concentration under reduced pressure, the residue was purified by column chromatography to give product E182 (32 mg, 0.070 mmol) in 83% yield. ^1^H NMR (600 MHz, CDCl_3_) δ (ppm) 6.92-6.88 (m, 3H), 6.86-6.84 (m, 2H), 6.83-6.81 (dd, 1H, J = 8.2, 2.0 Hz), 4.75 (d, 1H, J = 4.5 Hz), 4.74 (d, 1H, J = 4.5 Hz), 4.27-4.22 (m, 2H), 4.03 (t, 2H, J = 6.2 Hz), 3.90 (s, 3H), 3.90-3.88 (m, 2H), 3.87 (s, 3H), 3.13-3.07 (m, 2H), 2.45 (t, 2H, J = 7.2 Hz), 1.92-1.87 (m, 2H), 1.87-1.81 (m, 2H), 1.40-1.30 (m, 2H), 0.95-0.85 (m, 2H). ^13^C NMR (150 MHz, CDCl_3_) 177.78, 149.68, 148.00, 146.73, 145.28, 133.87, 132.93, 118.99, 118.33, 114.30, 113.06, 109.86, 108.66, 85.90, 85.78, 71.71, 68.70, 56.06, 55.98, 54.18, 54.15, 28.44, 21.58.

**E188**

To a stirred solution of E182 (11 mg, 0.024 mmol) and E175 (16 mg, 0.024 mmol) in anhydrous DMF (1.000 ml) at room temperature was added PyBOP (15 mg, 0.029 mmol), followed by the addition of DIPEA (13 µL, 0.072 mmol). The resulting mixture was stirred for 12h. After concentration under reduced pressure, the residue was purified by column chromatography to give 18 mg product E188 in 72% yield. ^1^H NMR (600 MHz, CD_3_OD) δ (ppm) 6.99 (d, 1H, J = 1.2 Hz), 6.95 (d, 1H, J = 1.8 Hz), 6.93-6.89 (m, 2H), 6.81 (dd, 1H, J = 8.4, 1.8 Hz), 6.77 (d, 1H, J = 8.1 Hz), 4.73 (d, 1H, J = 4.2 Hz), 4.71 (d, 1H, J = 4.2 Hz), 4.35 (dd, 1H, J = 8.4, 5.4 Hz), 4.26-4.22 (m, 2H), 4.04-3.97 (m, 2H), 3.87-3.86 (m, 1H), 3.86 (s, 3H), 3.85 (s, 3H), 3.60-3.57 (dd, 1H, J = 11.4, 5.4 Hz), 3.54-3.49 (m, 2H), 3.35 (s, 6H), 3.28-3.20 (m, 2H), 3.16-3.12 (m, 2H), 2.99 (s, 6H), 2.47-2.35 (m, 4H), 1.88-1.77 (m, 5H), 1.72-1.57 (m, 3H). ^13^C NMR (150 MHz, CD_3_OD) 174.87, 172.83, 156.28, 149.53, 148.03, 147.75, 145.95, 134.14, 132.41, 118.65, 118.46, 114.70, 113.15, 110.09, 109.64, 86.09, 85.90, 72.46, 71.33, 71.21, 68.79, 63.05, 55.17, 55.05, 54.04, 53.96, 52.74, 48.45, 41.49, 37.07, 35.04, 28.70, 28.21, 25.02, 22.39.

**2. Supplementary Figures**

**
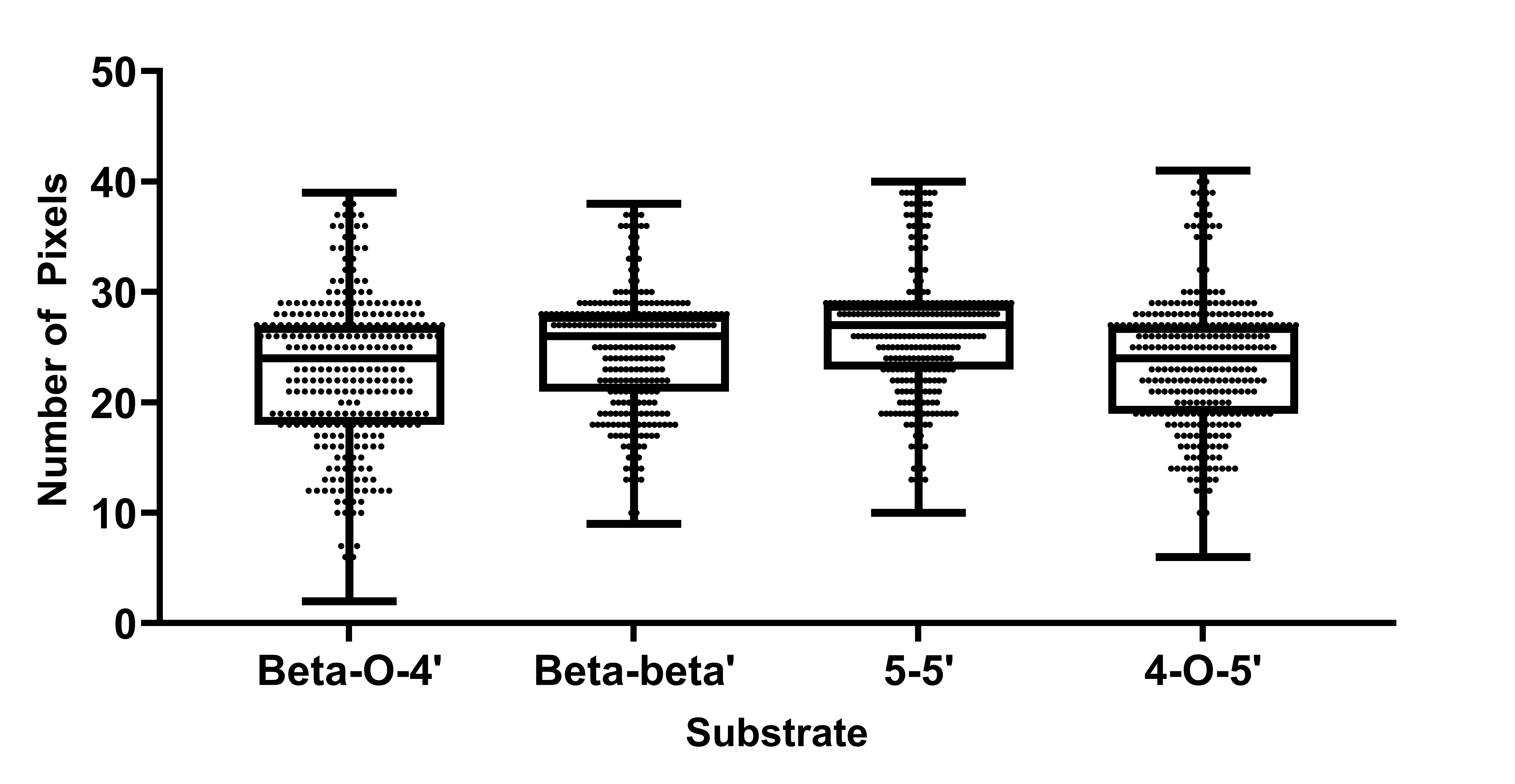
**

**Supplementary Figure S1.** Number of pixels per sample spot for 1,150 samples. Number of pixels were counted after empty pixels (i.e., pixels that lacked signals with a relative signal intensity of approximately ten times above background noise) were excluded.


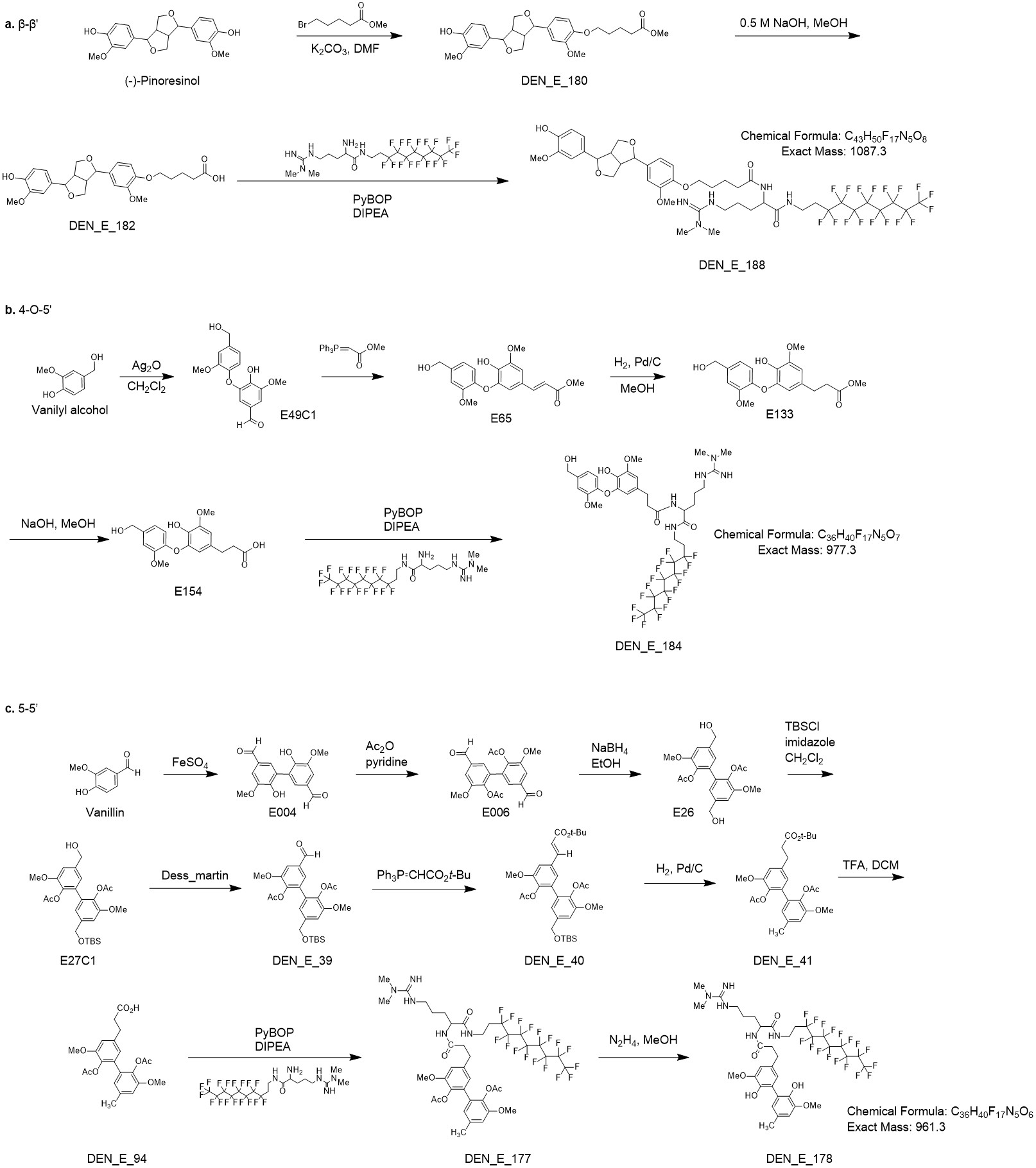


**Supplementary Figure S2.** Synthesis scheme of NIMS-tagged **a** ꞵ-ꞵ', **b** 4-O-5’, and **c** 5-5’ substrates.


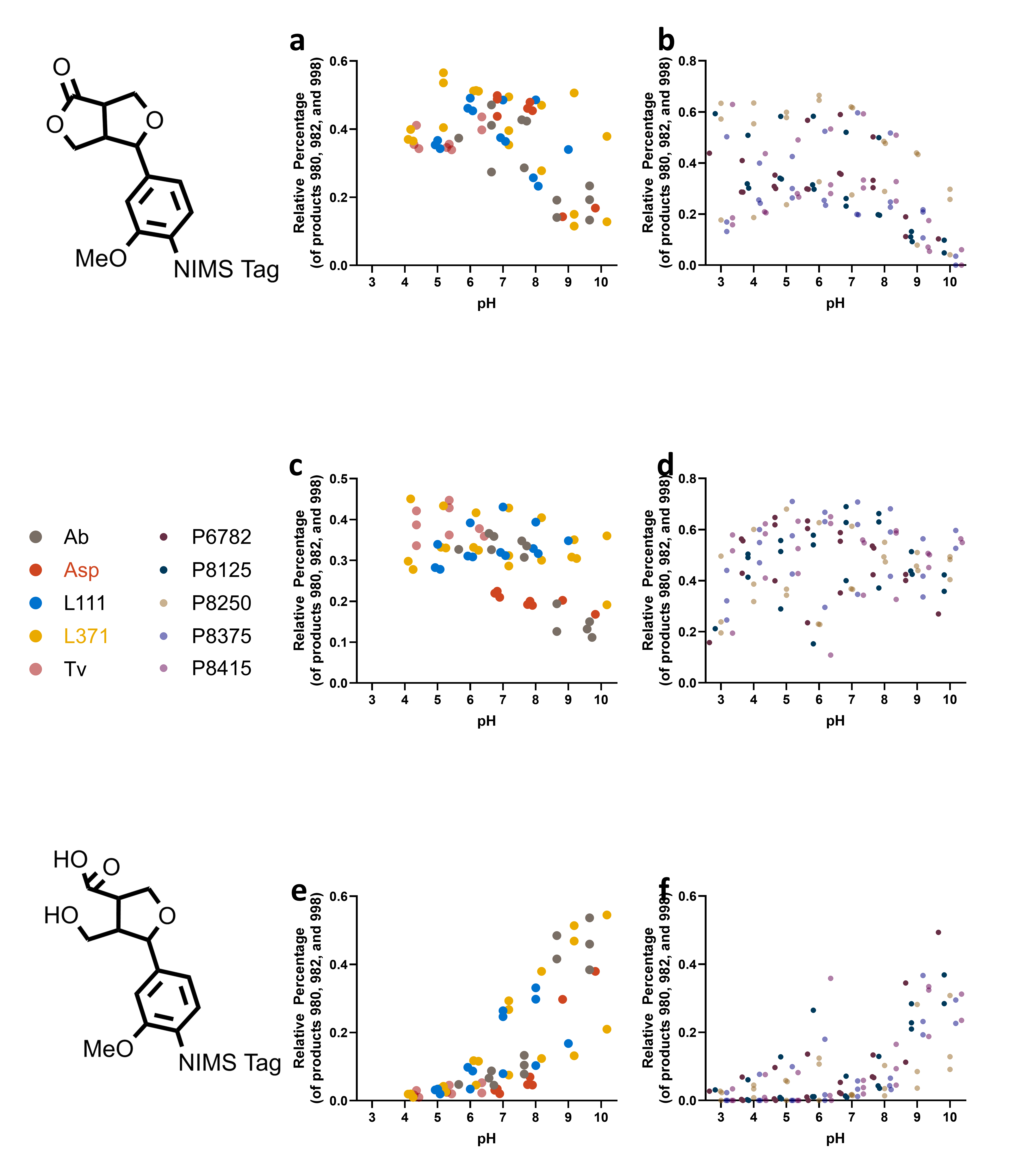


**Supplementary Figure S3.** Average relative proportions of products from ꞵ-ꞵ substrate cleavage. Statistical correlation between pH and relative products were determined via Pearson correlation (two-tailed). **a** 980 m/z product relative proportion in laccase reactions (P<0.0001), **b** 980 m/z product relative proportion in horseradish peroxidase reactions (P<0.0001), **c** 982 m/z product relative proportion in laccase reactions (P<0.0001), **d** 982 m/z product relative proportion in horseradish peroxidase reactions (P=0.1576), **e** 998 m/z product relative proportions in laccase reactions (P<0.0001), and **f** 998 m/z product relative proportions in horseradish peroxidase reactions (P<0.0001).


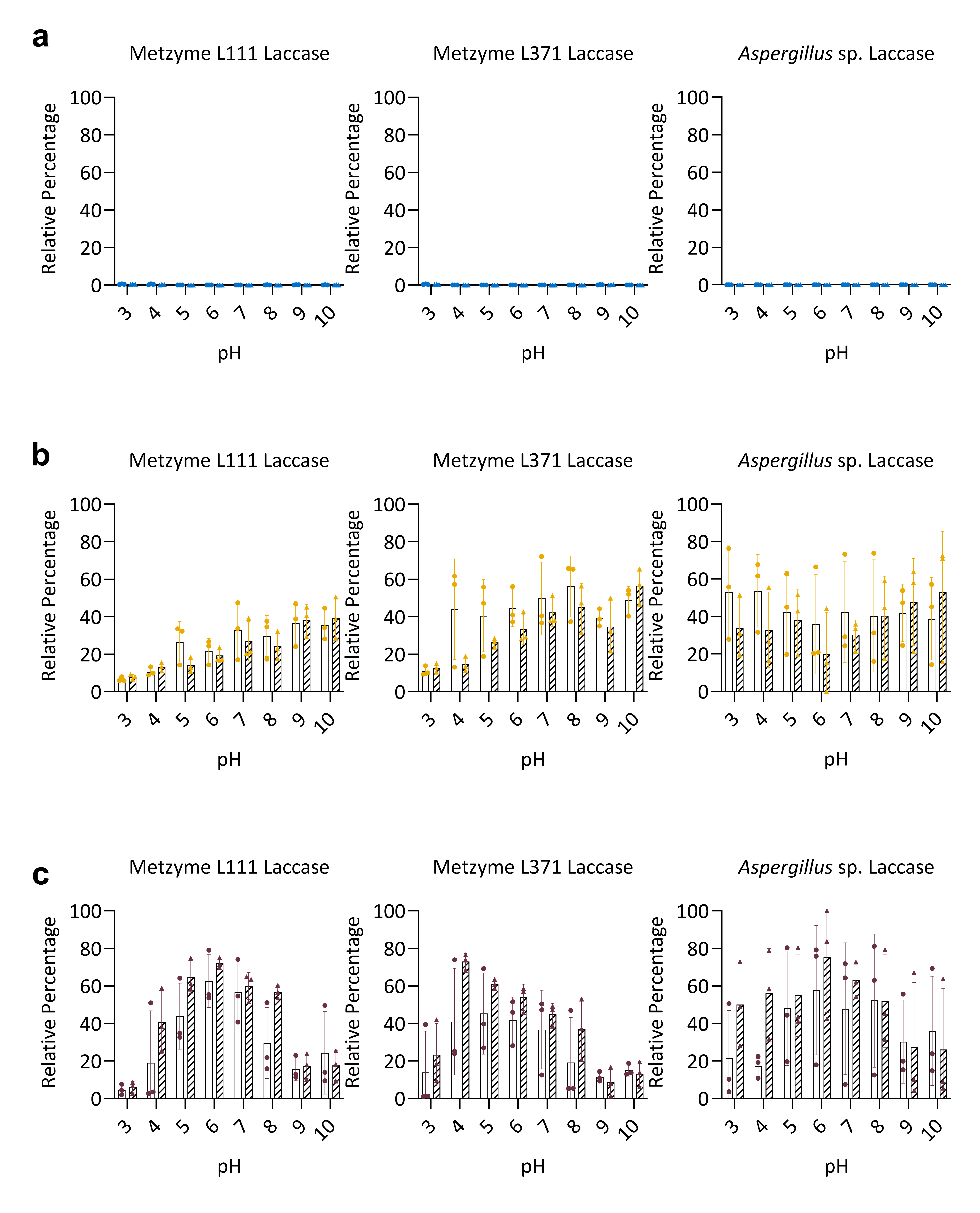


**Supplementary Figure S4.** Comparison of product profiles for the 5-5’ substrate tested with (hashed bars) and without 1-hydroxybenzotriazole as the mediator (white bars). Blue indicates cleaved products, yellow indicates modified products, and burgundy indicates polymerized products. No significant differences were detected between mediator and no-mediator treatments for each pH point (two-way ANOVA, Šídák’s multiple comparisons test, with a single pooled variance).


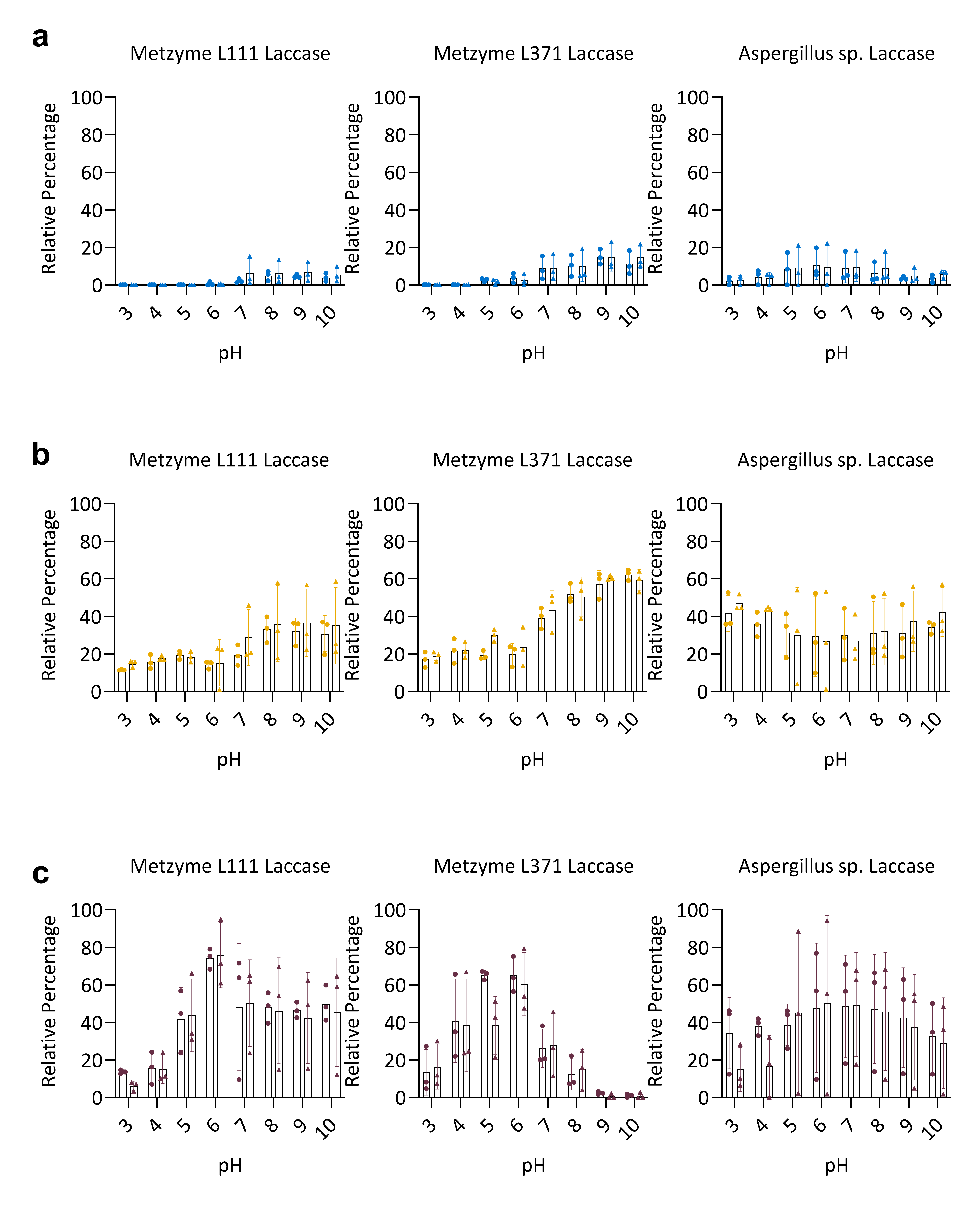


**Supplementary Figure S5.** Comparison of product profiles for the 4-O-5’ substrate tested with (hashed bars) and without 1-hydroxybenzotriazole as the mediator (white bars). Blue indicates cleaved products, yellow indicates modified products, and burgundy indicates polymerized products. No significant differences were detected between mediator and no-mediator treatments for each pH point (two-way ANOVA, Šídák’s multiple comparisons test, with a single pooled variance).

**
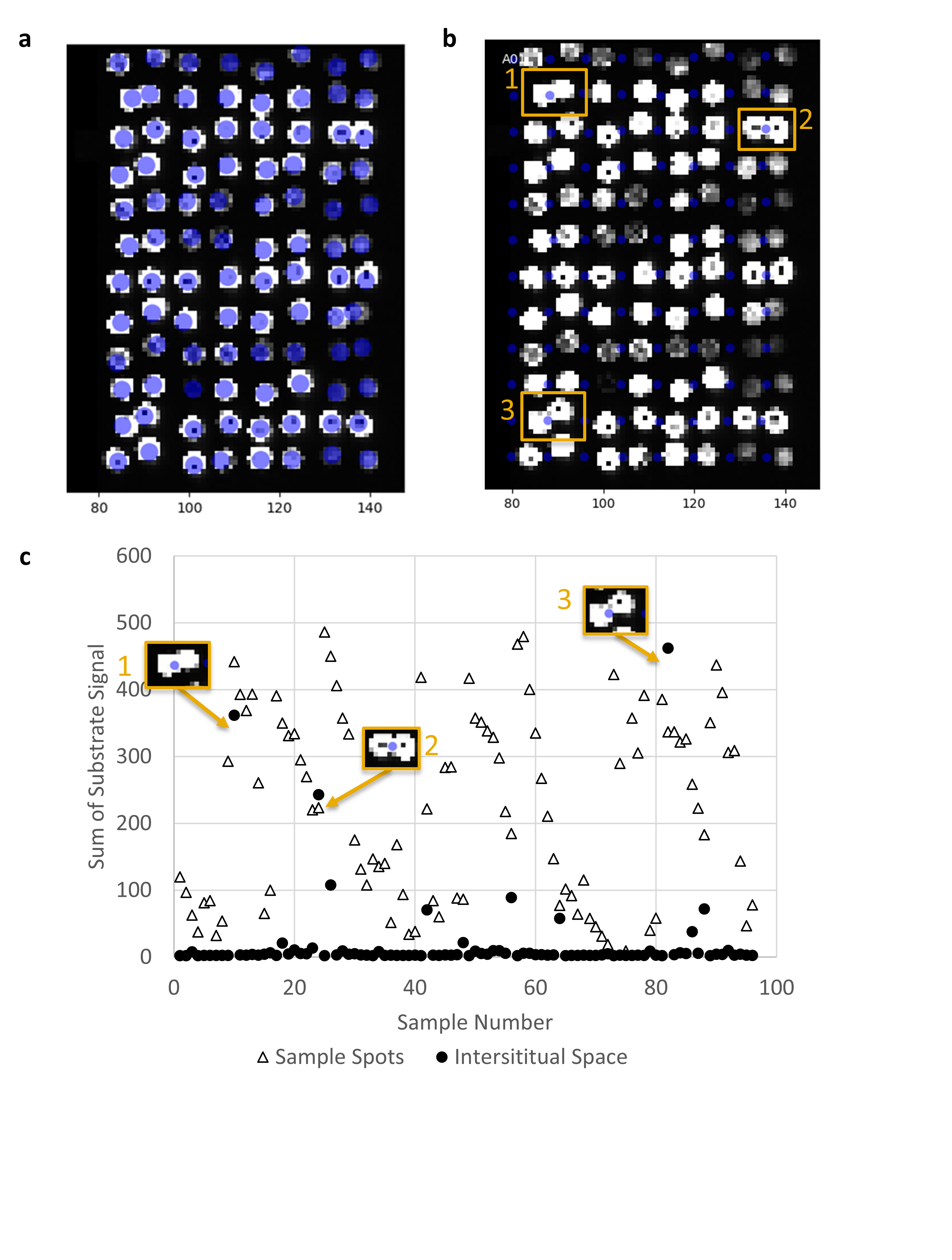
**

**Supplementary Figure S6.** Signal intensity of sample substrates (1088.3 *m/z*, 1151.3 *m/z*, 962.3 *m/z*, and 978.3 *m/z*) on sample spots and in between samples spots containing substrates and no enzymes. **a** OMAAT mask placed over sample spots (mask spots covered 10-14 pixels). **b** OMAAT mask placed over the interstitial space (mask spots covered 2-4 pixels). **c** Sum of signal intensities (the signal intensities for all pixels in a given mask spot were averaged, then the average intensity for each of the four ions was summed). Insets indicate interstitial spaces that were contaminated due to overlapping samples. Approximately 2% of all sample spots (46 out of 2048) appeared overlapping by visual inspection.

1. Mouterde LMM, Flourat AL, Cannet MMM, Ducrot PH, Allais F. “Chemoenzymatic total synthesis of a naturally occurring (5-5′)/(8′-O-4″) dehydrotrimer of ferulic acid.” Eur J Org Chem, 2013: 173-179. [↑](#endnote-ref-1)
2. JCS Perkin Trans 1 1994, 3485-3498 Ralph, J., S. Quideau, J.H. Grabber and R.D. Hatfield. “Identification and synthesis of new ferulic acid dehydrodimers present in grass cell walls.” J. Chem. Soc., Perkin. Trans 1, 1994, 3485-3498. [↑](#endnote-ref-2)
